# Duffy blood group gene polymorphisms among malaria vivax patients in four areas of the Brazilian Amazon region

**DOI:** 10.1186/1475-2875-6-167

**Published:** 2007-12-19

**Authors:** Carlos E Cavasini, Luiz C de Mattos, Álvaro AR D'Almeida Couto, Vanja SC D'Almeida Couto, Yuri Gollino, Laurence J Moretti, Cláudia R Bonini-Domingos, Andréa RB Rossit, Lilian Castilho, Ricardo LD Machado

**Affiliations:** 1 Centro de Investigação de Microrganismos, Faculdade de Medicina de São José do Rio Preto. Av. Brigadeiro Faria Lima, 5416. São José do Rio Preto, São Paulo State 15090-000, Brazil; 2 Imunogenetics Laboratory, Department of Molecular Biology, Faculty of Medicine from São José do Rio Preto, São José do Rio Preto, São Paulo State, Brazil; 3 SEAMA Faculty, Macapá, Amapá State, Brazil; 4 Evandro Chagas Institute, Malaria Program, Belém, Pará Sate, Brazil; 5 Hemoglobin Diseases Laboratory, IBILCE/UNESP, São José do Rio Preto, São Paulo State, Brazil; 6 Blood Bank, Campinas University, Campinas, São Paulo State, Brazil

## Abstract

**Background:**

Duffy blood group polymorphisms are important in areas where *Plasmodium vivax *predominates, because this molecule acts as a receptor for this protozoan. In the present study, Duffy blood group genotyping in *P. vivax *malaria patients from four different Brazilian endemic areas is reported, exploring significant associations between blood group variants and susceptibility or resistance to malaria.

**Methods:**

The *P. vivax *identification was determined by non-genotypic and genotypic screening tests. The Duffy blood group was genotyped by PCR/RFLP in 330 blood donors and 312 malaria patients from four Brazilian Amazon areas. In order to assess the variables significance and to obtain independence among the proportions, the Fisher's exact test was used.

**Results:**

The data show a high frequency of the *FYA/FYB *genotype, followed by *FYB/FYB, FYA/FYA*, *FYA/FYB-33 *and *FYB/FYB-33*. Low frequencies were detected for the *FYA/FY*^*X*^, *FYB/FY*^*X*^, *FYX/FY*^*X *^and *FYB-33/FYB-33 *genotypes. Negative Duffy genotype (*FYB-33/FYB-33*) was found in both groups: individuals infected and non-infected (blood donors). No individual carried the *FY*^*X*^*/FYB-33 *genotype. Some of the Duffy genotypes frequencies showed significant differences between donors and malaria patients.

**Conclusion:**

The obtained data suggest that individuals with the *FYA/FYB *genotype have higher susceptibility to malaria. The presence of the *FYB-33 *allele may be a selective advantage in the population, reducing the rate of infection by *P. vivax *in this region. Additional efforts may contribute to better elucidate the physiopathologic differences in this parasite/host relationship in regions endemic for *P. vivax *malaria, in particular the Brazilian Amazon region.

## Background

*Plasmodium vivax *has been the most common cause of the human malaria parasite in the Brazilian Amazon region over the last seven years [1]. Innate resistance to malaria infections in humans is conferred by different blood group polymorphisms. The association of the Duffy blood group (*FY*) with *P. vivax *human malaria has been well-documented, where Duffy-negative individuals are naturally resistant to invasion by this parasite [2].

The Duffy blood group antigen has been identified as a scavenger on the red blood cell (RBC) surface eliminating excesses of circulating toxic chemokines, named Duffy antigen/receptor for chemokines (DARC) [3]. The determinant, a glycoprotein that passes through the membrane seven times, includes a 35–43 KDa epitope in the extracellular, N-terminal domain that mediates erythrocyte invasion by *P. vivax *merozoites [4]. The *Fy *gene has two exons (Fy^a ^and Fy^b^) that are encoded by the co-dominant alleles *FYA *and *FYB*, located on chromosome 1 [5,6]. The corresponding anti-Fy^a ^and anti-Fy^b ^antibodies define four different phenotypes; Fy(a+b+), Fy(a+b-), Fy(a-b+) and Fy(a-b-) [7]. The *FYA *and *FYB *alleles differ by a point mutation in the major cDNA transcript [8], encoding glycine in Fy^a ^or aspartic acid in Fy^b ^at residue 42 of the most important form of the protein, encoded by the exon 2 [9,8]. The molecular mechanism that gives rise to the null Duffy phenotype [Fy(a-b-)] has been classically associated with a point mutation in the GATA box of the DARC promoter which silences the gene encoding the Duffy system antigens in the RBCs of these individuals resulting in a *FYB *allele [9]. Recently, the *FYA *allele has been identified [10] and a new *FYB *allele was found with three single nucleotide polymorphisms (11). It has been demonstrated in blood donors from Southeast Brazil that Caucasians and African descendents were serologically Fy(b-) with the majority of the African descendents being *FYB *with the GATA single nucleotide polymorphism (SNP), while the majority of Caucasian typing Fy(b-) had *FYB *with 265T/298A SNPs [11]. The Fy-negative is common on RBCs of Negro individuals from ethnic African groups, but it is rare in many other populations [12].

Previous studies have demonstrated that heterozygous Duffy negative malaria individuals remain susceptible to infections by *P. vivax *[13,14]. It was demonstrated in individuals living in a malaria endemic region of Papua New Guinea that Duffy binding protein adherence to RBCs is significantly reduced for erythrocytes of *P. vivax *malaria patients who carry one Duffy-negative allele [15]. In addition, it was observed that in different areas from the Brazilian Amazon region some of the Duffy phenotypes showed significant correlation between blood donors and malaria patients [16]. On the other hand, a previous study in the Western Brazilian Amazon region (Rondônia State) did not correlate *Fy *heterozygosity with protection against *P. vivax *[17]. The present study reports the Duffy blood group genotyping in *P. vivax *malaria patients from four different Brazilian endemic areas, exploring these significant phenotypic associations between blood group variants, as well as polymorphic regions of RBC receptors, with susceptibility or resistance to malaria.

## Methods

### Study population

The study took place from May 2003 to August 2005. The vivax malaria patients (n = 312) who were enrolled in this study complied with the following criteria: they sought medical assistance presenting clinical malaria symptoms, were over 18 years old and had positive results for thick blood film/molecular diagnosis. A control group consisted of blood donors (n = 330) and according to the Brazilian blood bank policy they complied with the following criteria: they were over 18 years old, of both genders and belonging to all blood groups. Additionally, their place of birth was in the study area, they reported never suffering from malaria attacks and had no signs of malaria during the initial interview and had negative results for thick blood film/molecular diagnosis. The controls were matched to the patients in respect to age (± 5 years), gender and ethnicity. All of the control subjects were genetically independent. Blood samples were collected from all participants (Macapá/Amapá State, Belém/Pará State, Porto Velho/Rondônia State and Rio Branco/Acre State), after informed consent.

### Genomic DNA extraction, PCR amplification and RFLP analysis

The Duffy blood group genotypes were assessed using PCR/RFLP with modifications as described previously [11]. Briefly, the DNA was extracted from frozen pellets of infected erythrocytes using the Easy-DNA™ extraction kit (Invitrogen, California – USA). The PCR was performed with 100 ng of DNA, 50 pmol of each primer, 2 nmol each dNTP, 1.0 U Taq DNA polymerase, and buffer, in a total volume of 50 μL. The promoter region was amplified using the FYN1 and FYN2 primers that flank the GATA box motif. To determine the Duffy RBC polymorphism, FYAB1 sense, and FYAB2 reverse sense primers were used [18]. The amplification conditions were performed as described by Castilho *et al *[11]. PCR products were run on 1.5% agarose gel, followed by ethidium bromide staining and photo-documentation using a Gel Doc 1000 (BioRad, Town, USA). The RFPL analysis was digested during three hours with *Ban*1, *Msp*A1 and *Sty*1 restriction enzymes as previously described [11].

### Statistical analysis

In order to access the significance of the variables and to obtain independence among the proportions, the Fisher's exact test was used. The mean ages of patients and controls were 29 years (± 14 SD) and 28 years (± 8 SD), respectively. All the studied groups showed no statistically significant differences in mean ages or ethnicity, indicating well-matched populations. The same results were obtained when we compared the two different groups from each area.

## Results

The genotypic and allelic frequencies of the Duffy blood group in the 330 blood donors and 312 patients infected by *P. vivax *as determined by PCR/RFLP are summarized in Table 1. The data show a high frequency of the *FYA/FYB *genotype in 199 individuals (31.0%) of the studied population. This is followed by 117 (18.2%) and 94 (14.6%) of homozygotes for the *FYB *and *FYA *alleles, respectively. While the frequencies for heterozygote individuals with *FYA/FYB-33 *and *FYB/FYB-33 *were comparable at 14.9% (96). Low frequencies were detected for the *FYA/FY*^*X*^, *FYB/FY*^*X*^, *FYX/FY*^*X *^and *FYB-33/FYB-33 *genotypes. The negative Duffy genotype (*FYB-33/FYB-33*) was found in both individuals infected by *P. vivax *and unaffected blood donors.

**Table 1:** Genotyping results of the Duffy Blood Group System in 642 individuals (donors and patients infected with *Plasmodium *viva*x*) from Brazilian Amazon region.

**Genotype result**	**nt 125 *FYA*/*FYB *Donors/Patients**	**nt 265 *FYB*^*WK *^or *FY*^*X*^**	**GATA (n) *FYB*^*ES *^or *FY0 *or *FYB-33***	**Predicted Phenotype Donors/Patients**
***FYA/FYA***	G/G 43/51	C/C	T/T	
***FYA/FY*^*X*^**	G/A 03/05	C/T	T/T	Fy(a+b-) n = 108/90
***FYA/FYB-33***	G/A 62/34	C/C	T/C	
				
***FYA/FYB***	G/A 90/109	C/C	T/T	Fy(a+b+) n = 90/109
				
***FYB/FYB***	A/A 54/63	C/C	T/T	
***FYB/FY*^*X*^**	A/A 06/04	C/T	T/T	Fy(a-b+) n = 114/109
***FYB/FYB-33***	A/A 54/42	C/C	T/C	
				
***FY*^*X*^/*FY*^*X*^**	A/A 00/02	T/T	T/T	Fy(a-b-) n = 18/04
***FYB-33/FYB-33***	A/A 18/02	C/C	C/C	
				
Total	330/312			

Table 2 shows a comparison of the results of genotyping and inferred phenotypes of *FY *among blood donors and patients infected by *P. vivax *in the Amazon region. In respect to the allelic combinations of *FY*, there was a significant difference between donors and patients only for the *FYB-33/FYB-3 *genotype (P = 0.0003). As for the heterozygotes, the results demonstrated a significant difference (P = 0.0404) between those with the *FYA/FYB *genotype, which was lower for blood donors (90 - 27.27%) than for patients infected by *P. vivax *(109 - 34.93%). No individuals were identified with the *FY*^*X*^*/FYB-33 *genotype.

**Table 2:** Comparison of genotyping results and allele frequencies of the Duffy Blood Group System among blood donors and patients infected with *P. vivax *from Brazilian Amazon region.

**Duffy blood group system**		**Brazilian Amazon Region**		
	
**Genotypes**	**Predicted Phenotype**	**Donors (n = 330)**	**Patients *P. vivax *(n = 312)**	***P***
***FYA/FYA***	Fy(a^+^b^-^)	43	51	0,2644
***FYA/FY*^*X*^**	Fy(a^+^b^-^)	3	5	0,4942
***FYA/FYB-33***	Fy(a^+^b^-^)	62	34	0,0055*
***FYA/FYB***	Fy(a^+^b^+^)	90	109	0,0404*
***FYB/FYB***	Fy(a^-^b^+^)	54	63	0,2208
***FYB/FY*^*X*^**	Fy(a^-^b^+^)	6	4	0,7530
***FY*^*X*^/*FY*^*X*^**	Fy(a^-^b^-^)	-	2	0,2358
***FYB/FYB-33***	Fy(a^-^b^+^)	54	42	0,3205
***FYB-33/FYB-33***	Fy(a^-^b^-^)	18	2	0,0003*
			
**Alelles**		**Alelles Frequencies**		
	
***FYA***		0,365	0,400	0,2896
***FYB***		0,390	0,450	0,0351
***FY*^*X*^**		0,014	0,021	0,5726
***FYB-33***		0,230	0,128	0,0000

* Fisher's Exact Test.

The frequency of individuals with the *FYB-33 *allele among blood donors was 35.15% versus 24.36% in patients. This difference was statistically significant (P = 0.0033). The prevalence of the *FYB *allele was significantly higher (P = 0.0358) among malaria-infected patients. However, significant differences were not detected in respect to the frequency of individuals with the *FY*^*X *^and *FYA *alleles in the two study groups (Table 2).

## Discussion

Thus far, innate resistance to malaria infections in humans has been attributed to blood group polymorphisms. Duffy blood group polymorphisms are important in areas where *P. vivax *predominates, because this molecule acts as a receptor for this protozoan (but not for the other human malaria parasites) on the surface of RBCs [19]. Field observations from West Africa and Ethiopia have indeed established a strong correlation between absence or low endemicity of *P. vivax *malaria and the high prevalence of the Duffy negative allele [20,21]. Little is known on the frequency of RBC polymorphisms that confer either partial or complete resistance against malaria. The data obtained in the present study emphasize the importance of the evaluation of Duffy blood group genotypes in malaria endemic areas of the Brazilian Amazon region.

The Brazilian population has a highly heterogeneous ethnic composition, a result of the hybridization of the numerous native indigenous populations and immigrants from Europe, Africa and Asia. The immigration flow was not uniform in the different regions of the country [22-24]. The differential distribution of Duffy antigenic determinants among ethnic groups is an aspect characteristic of this blood system. Hence, this has been used as a marker for the ethnic composition as well as an indicator in the populational evolution. In the current study, significant differences in the allelic frequencies of the *FY *gene were identified when compared with previous studies of patients infected with human plasmodium in Brazilian Amazon regions [17,25-27]. However, the *FYA*, *FYB *and *FYB-33 *alleles showed differential distributions compared to the population of the southeastern region of Brazil [11]. The genotypic compositions obtained in this study demonstrated significant variations compared to previous studies performed in the states of Pará and Rio Grande do Sul [28,29] and in the cities of São Paulo [30], Campinas [11] and Ribeirão Preto [31] all of which are in the state of São Paulo, Southeastern region of Brazil.

The currently obtained results showed that the *FYA/FYB *genotype was the most common, followed by the homozygotes for the *FYB *and *FYA *alleles and by the heterozygotes *FYA/FYB-33 *and *FYB/FYB-33 *(Table 1). The *FYB-33/FYB-33 *genotype, which is classically seen in individuals unaffected by *P. vivax *infection, was identified in 3.2% of the general population and in 5.5% of blood donors. These numbers differ from previous reports on a group of malaria patients uninfected by *P. vivax *in the state of Rondônia [17], where the frequency of this genotype was 12% and also for a mixed sample from the Northern and Southeastern regions of Brazil [27]. Some populations had higher genotypic frequencies for *FYA/FYB *and *FYB/FYB-33 *than for a caucasian-like and negroid population from the Southeastern of Brazil [11]. On the other hand, the frequency of the *FYB-33/FYB-33 *genotype was lower. In fact, with the exception of negroid ethnic groups, this genotype is extremely rare [12,30,32,33].

The *FYA *allele is common in European and Oriental peoples but it is rare in African Negroes. Additionally, the *FYB *is more common in populations classified as white than in asiatic and negroid populations [34,35]. The frequency of the *FYA *and *FYB *alleles in the Brazilian Amazon population was higher than those in the Southeastern region [11] probably due to the massive influence of Portuguese colonization in the North region of Brazil as well as the presence of Amerindians [36], as recent molecular analysis has corroborated that Amerindians have an Asian origin [37]. Indeed, in the North region of Brazil, studies carried out with different tribes of Amerindians have shown that the *FYA *allele is the most common [31,38]. As expected, a lower frequency of the *FYB-33 *allele was observed in the North region (P = 0.0001). Although a high prevalence of this allele was demonstrated in isolated communities in the states of Amapá and Pará, North region of Brazil [39], the introduction of a negroid ethnic component in the Amazon region is recent [40,41]. In respect to the *FY*^*X *^allele, there does not seem to be a differential ethnic distribution as, the frequency detected here is similar to those described in other Caucasian-like populations in Brazil [11] and also in Europe [42].

The different *FY *allelic frequencies in individuals from North compared to Southeastern Brazil, may be due to the contribution of the three major ethnic groups (Europeans, in particular Portuguese; Blacks and Amerindians) in the formation of both populations. The Amerindian contribution was higher in the north of the country whereas European migration took place more in the South and Southeastern regions [43-45]. In spite of the current knowledge of the relationship between structure/function and tissue location of DARC, the functional significance of each of the alleles and the different genotypic combinations require further elucidation. The variation in the ethnic composition of the urban and rural populations of the Brazilian Amazon region and of distinct regions in Brazil [26,46], may influence the allelic and genotypic distributions reflecting in matrixes of genetic mechanisms favorable to the susceptibility to infectious and parasitic diseases, in particular to malaria.

The frequency of the allele with the *FY *GATA mutation (*FYB-33*) was greater in blood donors than in patients infected with malaria (P = 0.0033), suggesting that there is a reduction in the infection rate of carriers of the *FYB-33 *allele. These results were recently reinforced by data from of individuals infected by *P. falciparum *when compared to those infected by *P. vivax *in Brazilian Amazon regions [27]. The *FYB-33 *allele is a variant of the *FYB *allele resulting from a T → C point mutation in the gene promoter region (neucleotide-33), which abolishes its expression [9]. The same occurs in the *FYA *allele, determining the *FYA*^*null *^allele [10]. The presence of the *FYB-33 *allele results in a 50% reduction in the Duffy protein expression on the erythrocyte surface [15,47]. This process demonstrates the action of the dose-related effect of the gene [15,48], which may limit the invasion process of red blood cells by the parasite, although susceptibility to *P. vivax *may occur in heterozygotic Duffy-negative individuals [13,14]. Thus the *FY/FYB-33 *genotypic combination, with either Fy(a+b-) or Fy(b+b-), seems to convey a reduction in the susceptibility to malaria. *In vitro *studies [15] support this hypothesis as RBCs that express these phenotypes have a significant reduction in cytoadherence of the parasite when compared to RBCs that express Fy(b+b+). Recently, in Papua New Guinea, a significant reduction in infection by *P. vivax *was observed in Duffy negative individuals heterozygotic for the *FYA*^*null *^allele [49].

In this study a low frequency of the negative Duffy genotype (*FYB-33/FYB-33*) was detected among uninfected subjects. As has previously been demonstrated, the absence of the Fy antigen in many ethnic Negro groups and their descendents, does not seem to exert any deleterious effect, however it does bestow a natural resistance against infection by *P. vivax *[2,9,14,35,50,51]. These individuals are homozygotes for the *FYB *mutation in the GATA, that completely abolishes the Fy expression in erythrocytes but not in cells of other tissues [9]. Recent reports have provided evidence on the transmission among Duffy-negative patients in Africa [52] and in Brazil [53]. These data suggest the possibility that *P. vivax *is utilizing alternative receptors, apart from Fy, for binding in the erythrocyte invasion process. Whether *FYB *carrying the GATA box mutation is the primordial gene that encodes the Duffy system antigens or whether the GATA box mutation has evolved to escape malaria infection per se is controversial. However, the presence of this allele (*FYB-33*) may be a demonstration of the selection advantage by mutation in the population. Thus, in the Brazilian Amazon, where *P. vivax *predominates, the frequency of the *FYB-33 *allele is higher than expected given the ethnic population, both in terms of heterozygotes and homozygotes. This might, over time, cause an increase in the number of Duffy-negative individuals in the population and, as a consequence, a reduction in the rate of infection by *P. vivax *in this region.

In the group of patients infected by *P. vivax*, different allelic combinations with *FY*^*X *^were detected, including five (1.6%)*FYA/FY*^*X *^individuals, four (1.3%) *FYB/FY*^*X *^and two (0.6%) *FY*^*X*^*/FY*^*X *^(Table 2). The frequency of this allele among patients and blood donors did not show statistical differences (P = 0.3873). Studies carried out in blood samples from European and American Caucasians and Afro-American individuals, demonstrated that the C265T (*FY*^*X*^) mutation altered the Duffy protein expression on the RBC surface differently, depending on the ethnic group, using at least two mechanisms. One mechanism involves silent transcription in one of the *FYB *alleles and the other affects the translation and/or the stability of the protein [47,54]. These studies showed that heterozygote individuals for the *FY*^*X *^(*FY*^*WK*^) allele have 50% less of the Duffy protein on the surface of the membrane of the erythrocyte. On the other hand, individuals homozygotic for the *FY*^*X *^allele have about one tenth of the antigen expression in the erythrocytes [47]. However, the information shown by the present study suggest that these polymorphisms do not seem to be associated with susceptibility to malaria.

As illustrated in Table 2, a higher frequency of the *FYA/FYB *genotype in patients compared to blood donors (P = 0.0404) was detected. In principle, this seems to indicate that the condition of heterozygosis, resulting from the expression of the *FYA *and *FYB *genes, favors infection by *P. vivax*. In fact, as has already been demonstrated by us, based on a phenotyping study, Fy(a+b+) individuals may be more susceptible to infection by this species of *Plasmodium *[16]. Although no qualitative or quantitative measurements of the Duffy glycoprotein expressions were made in this study, we saw a larger number of malaria episodes among patients with the heterozygote genotype than the homozygote genotype. The basis of this observation has not been determined yet, but the phenotypic [16] and genotypic data presented from the same Brazilian Amazon areas associated with pertinent publications, point to the possibility that heterozygote individuals modulate the susceptibility to malaria by *P. vivax *by means of quantitative and/or qualitative variations that affect the Duffy antigen expression on erythrocytes. In the first aspect, *in vitro *studies demonstrate differences in the levels of the expression of FY glycoprotein on the surface of the reticulocytes of Caucasian-like and Afro-American individuals with the Fy(a+b+) erythrocytic phenotype [48]. These authors verified that individuals with *FYA *and *FYB *alleles expressed a lower quantity of DARC than heterozygotes. Hence, it is possible that heterozygote individuals have a greater repertory of receptors for the possible variations that occur in the parasite protein binder to the human erythrocytes.

The recent demonstration of polymorphisms in the Duffy binding protein in isolates of *P. vivax *from the Brazilian Amazon [55] seems to support the observed results. An association, between human receptor polymorphisms and variations in the parasite binders of *Plasmodium falciparum *that modulate susceptibility to malaria, was also demonstrated [19,56-60]. Hence, apart from the different levels of the expression, the specific conformation of the Fya and Fyb antigens may determine differences in the susceptibility to infection. Nevertheless, one of the possible consequences of differential susceptibility to *vivax *malaria could be modifications in allelic frequencies of *FYA *and *FYB *in populations exposed to *P. vivax*, the most prevalent species in the Brazilian Amazon region. If in fact this process occurs as a consequence of infection, it seems to be necessary, but not sufficient to eliminate heterozygote individuals, even if they are affected by repetitive episodes of malaria by *P. vivax*, as the infection is rarely severe and occurs in the entire age range with different frequencies for different ages and regions of the Brazilian Amazon without lethality [61,62].

Based on the significant associations described herein, our data differ from previous studies [17] carried out in the western Brazilian Amazon region as well as recent studies of patients from the Brazilian Amazon as a whole [27]. In respect to the first studies, these differences may be a result of the small sample sizes initially evaluated; the control group of the current study was composed of blood donors who reported never having experienced malaria against samples from individuals who had *P. falciparum *previously evaluated and the various ethnic groups in the different regions studied today (Eastern and Western Amazon). In relation to the recent studies, different to the findings observed in this study Brazilian Amazon population, the authors detected a significant association among infections by *P. vivax *and the *FYB/FYB *genotype, also conflicting with results of Wooley *et al *[48] in North American individuals (Caucasians and Afro-Americans). These divergences probably occurred due to the smaller sample size involved, and the fact that the control group consisted patients infected by *P. falciparum*.

## Conclusion

The Brazilian population presents an elevated degree of miscegenation which is implicated in variations in the allelic distribution of *FY*, which was also found in this study. Future analyses that consider haplotypic frequencies of the alleles and quantification of the DARC expression in populational groups in endemic and non-endemic areas, increasing the knowledge on the dynamics of this gene and its possible contribution as a modulator in the susceptibility to malaria. The data obtained in the present study supports the hypothesis that individuals with the *FYA/FYB *genotype have higher susceptibility to malaria. The presence of the *FYB-33*allele may be a selective advantage in the population, reducing the rate of infection by *P. vivax *in this region. However, the populational and longitudinal studies must be amplified accompanying groups of individuals with the *FYB-33 *allele and the *FYA/FYB *genotype, including clinical parameters, as well as the evaluation of their expressions which may contribute to better elucidate the physiopathologic differences in this parasite/host relationship in regions endemic for *P. vivax *malaria, in particular the Brazilian Amazon.

## Authors' contributions

CEC carried out all genotype assay. AADC, VSDC and CEC collected samples from *P. vivax *malaria individuals and blood donors. CEC, LCM, CRBD and LC participated in the design of the study and drafted the manuscript. RLM and ARBR conceived of the study and participated in all aspects of its design, acquisition of funding, execution, coordination and manuscript preparation. All authors read and approved the final manuscript.
